# Association between air flow limitation and body composition in young adults

**DOI:** 10.1186/s40101-021-00252-2

**Published:** 2021-01-19

**Authors:** Rodrigo Muñoz-Cofré, Pablo A. Lizana, Máximo Escobar Cabello, Claudio García-Herrera, Mariano del Sol

**Affiliations:** 1 Doctorado en Ciencias Morfológicas, Universidad de La Frontera, Temuco, Chile; 2 Centro de Excelencia en Estudios Morfológicos y Quirúrgicos, Universidad de La Frontera, Temuco, Chile; 3 Laboratory of Morphological Sciences, Instituto de Biología, Pontificia Universidad Católica de Valparaíso, Valparaíso, Chile; 4 Laboratorio de Función Disfunción Ventilatoria, Departamento de Kinesiología, Universidad Católica del Maule, Talca, Chile; 5 Departamento de Ingeniería Mecánica, Universidad de Santiago de Chile, Santiago, Chile

**Keywords:** Pulmonary function, Body composition, Body mass index, Obesity

## Abstract

**Background:**

Body composition (BC) influences respiratory system mechanics, provoking air flow limitation (AFL). The objective of this study was to determine the relationship of AFL in small- and medium-caliber airways with BC in young adults.

**Methods:**

Eighty-three individuals were recruited (40 men and 43 women). To determine AFL, the following measurements were taken: forced expiratory volume in the first second (FEV_1_), forced expiratory flow between 25 and 75% (FEF_25–75%_), airway resistance (Raw), and specific airway resistance (sRaw). The measured BC variables were body mass index (BMI), body fat percentage (%BF), and fat-free mass (FFM). Binary logistical regression analysis was used to estimate the association between the AFL variables and %BF, BMI, and %FFM, adjusting for weight and gender.

**Results:**

Among men, a relationship was observed between Raw and %BF (*r* = 0.728; *p* < 0.0001) and sRaw and BMI (*r* = 0.617; *p* < 0.0001). Among women, significant relationships were reported between Raw and BMI (*r* = 0.615; *p* < 0.0001) and sRaw and BMI (*r* = 0.556; *p* < 0.0001). Among participants with a BMI over 30 kg/m^2^, higher risks of increased Raw (OR = 26.8; *p* = 0.009) and sRaw (OR = 9.3; *p* = 0.002) were observed. Furthermore, higher %BF was associated with greater risks for increased Raw (OR = 14.04; *p* = 0.030) and sRaw (OR = 4.14; *p* = 0.028). In contrast, increased %FFM (OR = 0.14; *p* = 0.025) was a protective factor for lung function.

**Conclusion:**

Increased %BF is associated with increased AFL in small-caliber airways. Furthermore, increased %FFM is associated with decreased risk for Raw and sRaw in women. Therefore, evidence indicates that increased %FFM is a protective factor for adequate lung function.

## Background

Respiration is an essential function for survival; therefore, changes in pulmonary function can diminish quality of life and performance of daily tasks [1]. Various factors have been proposed to impact respiratory system mechanics, including body composition (BC) [2, 3]. Studies have been performed on the effects of muscle mass, lean body mass, and body fat on lung function, indicating its close relationship with forced vital capacity (FVC) and forced expiratory volume in the first second (FEV_1_) [4]. These relationships would indicate that changes in BC can impact medium-caliber airways [5, 6], a phenomenon that does not guarantee similar behavior in small-caliber airways.

The prevalence of obesity has increased alarmingly in developing countries. In Chile, the Organization for Economic Cooperation and Development (OECD) indicates that 74.2% of the population over 16 is overweight or obese. One of the most common measurements for evaluating obesity is body mass index (BMI). However, this measurement does not distinguish body composition components (fat mass, muscle mass, bone mass) [7, 8]. An increase or sudden excessive decrease in obesity has been negatively associated with alterations in FVC and FEV_1_. Furthermore, increased waist circumference, waist-hip ratio, and fat percentage have been linked with diminished lung function [2]. Thus, excessive fat accumulation alters the relationship between the lungs, thoracic wall, and diaphragm, diminishing pulmonary volume and consequently negatively impacting the cross-sectional diameter of the airways [4].

Fat tissue is the most variable BC component [9, 10]. Various risks are associated with the location and excess of fat tissue. Currently, mechanical and metabolic consequences have been reported for regional fat distribution, with particular importance placed on clinical evaluation and therapeutic behaviors to pursue after evaluation [11]. Furthermore, abdominal fat distribution has been largely associated with an increased risk for different cardiovascular diseases, type 2 diabetes, and acute myocardial infarction, among other diseases [2, 12], as well as lung function disorders [13].

Fat tissue can also act as an endocrine and paracrine organ by producing cytokines and bioactive mediators, promoting a proinflammatory state [14, 15]. A proinflammatory state is associated with lung hypoplasia, atopy, bronchial hyperresponsiveness, and increased asthma risk in obese individuals [13]. In this way, it has been reported that the association between mechanical disorders and airway inflammation increases air flow limitation (AFL). Regarding AFL, one of the most studied and easily accessed variables is FEV_1_. However, in subjects without a background of respiratory disease, it would be highly useful to include forced expiratory flow between 25 and 75% (FEF_25–75%_) and airway resistance (Raw) to detect respiratory problems early and avoid their chronic phase since they can deliver information on small-caliber airways [16].

AFL is defined as a maximum air flow reduction disproportionate with regard to the air flow a subject can displace from their lungs [17]. Under normal conditions, air flow is a function of Raw and pressure gradient (or conduction pressure). In turn, airflow resistance is the result of the area of the cross section of airways over lung volumes [16, 17]. Thus, AFL expresses the morphological state of small-scale airways. In this context, measuring FEV_1_, FEF_25–75%_ , and Raw will provide a comprehensive assessment of medium- to small-caliber airways and how they can be influenced by the BC of each subject.

In this context, the objective of this study was to determine the relationship of AFL in small- and medium-caliber airways with BC in young adult subjects.

## Materials and methods

### Participants

The following cross-sectional study was approved by the Ethics Committee at Universidad de Santiago de Chile (14/2020) and was conducted in accordance with The Code of Ethics of the World Medical Association (Declaration of Helsinki) for experiments involving humans. Upon inclusion, all participants received oral explanations about the objectives of the study, and informed written consent was obtained. To calculate the number of participants, the statistical program eNe 3.0 was used. Drawing on the research of Rodriguez et al. with a sample of 57 participants and a sRaw of 3.8 ± 1.03 cmH_2_O*s [18], a power of 80% and a significance level of 5% were determined. These parameters resulted in a figure of 36 participants of each gender; after factoring in a 10% drop-out rate, 40 were evaluated, which made the total number of participants 80. The inclusion criteria were as follows: being over 18 and under 30 years of age, having no signs of chronic and/or acute respiratory disease and presenting normal spirometry values (FEV_1_ > 80% predicted). Participants were excluded if they had a tobacco habit or if they had morphological alterations in the thorax or spinal column. Sampling was performed in March 2020. All study participants were evaluated in the Lung Function Laboratory at the Universidad Católica del Maule-Chile in a single session during the morning.

### Anthropometry

Height was measured with a SECA® anthropometer (model 220, Hamburg, Germany), recording the distance from floor to the vertex. The subject had to stand upright, with their heels together and their feet at a 45° angle. Heels, gluteals, back, and the occipital region were in contact with the anthropometer surface. Measurement was performed during maximum inhalation while the participant maintained their head in the Frankfurt plane. Body mass was measured with a SECA® scale (model 840, Hamburg, Germany) [19]. BMI was obtained by dividing weight in kilograms by height in square meters (kg/m^2^). The BMI standards were as follows: underweight < 18.5 kg/m^2^, normal weight between 18.5 kg/m^2^ and 25 kg/m^2^, overweight between 25 kg/m^2^ and 30 kg/m^2^, and obesity over 30 kg/m^2^ [20].

### Body composition

To evaluate BC, a bioelectric impedance device was used (TANITA MC-780 MA, Tanita Corporation, Tokyo, Japan). For the measurement, each participant was asked to wear no metallic objects and prior to the evaluation to drink no alcohol for 48 hours, not perform intense exercise for 12 h, not eat or drink (especially caffeine or diuretics) for 4 h, and be sure to urinate immediately before the evaluation [21]. The variables analyzed were weight, body fat percentage (%BF), and fat-free mass percentage (% FFM).

### Lung function

#### Spirometry

Spirometry was carried out with a Mediagraphics body plethysmograph (Platinum Elite DL® model, St. Paul, MN, USA). The highest FVC value was recorded out of three attempts meeting the acceptability and reproducibility criteria set by the American Thoracic Society (ATS). The variables used were FEV_1_ and FEF_25–75%_ [22].

#### Breathing volumes

Lung volume tests were performed with a Mediagraphics body plethysmograph (Platinum Elite DL® model, St. Paul, MN, USA). After cabin closure, the subject was instructed to take four normal breaths. The cabin was closed and it was indicated to perform four ventilations at current volume. Subjects were subsequently instructed to “pant softly,” attempting to move volumes between 50 and 60 mL while blocking their cheeks with their fingertips to avoid mouth pressure fluctuation. The panting frequency had to be approximately 60 per minute (1 Hz). The professional in charge activated the shutter for 2–3 s, after which maximum inhalation was indicated, followed by exhalation to residual volume (RV). Measurements were performed according to ATS norms [23]. The variables used were Raw and sRaw.

#### Maximum inhalatory and exhalatory pressure (MIP and MEP)

Participants’ MIP was measured by directing them to perform maximum exhalation, followed by blocking the pneumotachograph and requesting maximum inhalation against the closed valve. Participants’ MEP was evaluated by directing them to perform maximum inhalation, followed by blocking the pneumotachograph and requesting maximum exhalation against the closed valve. In both tests, the best result was selected from a minimum of three acceptable and reproducible maneuvers according to ATS norms [24].

### Statistical analysis

Descriptive statistics are used to summarize the data. The statistical program STATA 16 was used (StataCorp. Stata Statistical Software, College Station, TX: StataCorp. LP, USA). The normality of data was evaluated using the Shapiro-Wilk’s normality test. To evaluate differences in anthropometric, BC, and lung function variables between men and women, Student’s *t* test or Mann-Whitney *U* test was used for independent samples. To establish a correlation between BC and lung function, depending on the data distribution, the Pearson or Spearman *r* test was used. The 75th percentile (p75) of the %BF variable and the obese BMI cutoff (BMIO) were used as cutoff points for data dichotomization. Thus, p75 was used for Raw and sRaw, and the 25th percentile (p25) was used for FEV_1_ and FEF _25–75%_. Binary logistic regression analysis was performed to estimate the association between a high %BF (men > 25; women > 35) and BMIO (> 30 kg/m^2^). For lung function, the high Raw figure was > 1.45 for men and > 1.86 for women. The sRaw number for both genders was > 4.76. Low FEV_1_ was < 4.04 for men and < 3.09 for women. For FEF_25–75%_, the figures were < 3.98 for men and < 3.02 for women, adjusted for age and gender. To verify the model adjustment precision, the Hosmer-Lemeshow test was applied. The statistical significance level was established at *p* < 0.05.

## Results

The participants’ BC and anthropometric characteristics are shown in Table 1. The sample presented significant differences in weight (*p* < 0.0001) and height (*p* < 0.0001) by gender. Comparisons by gender showed no significant differences in BMI. The %BF (30.01 ± 5.63) was significantly higher among women than among men (*p* < 0.0001; *p* = 0.018).

**Table 1:** Anthropometric and body composition characteristics of the total sample and by gender

	Total sample	Male	Female	*p* value
Number (*n*/%)	83/100	40/48.19	43/51.81	–
Age (years)	21.66 ± 2.22	21.93 ± 2.73	21.42 ± 1.60	0.421^MW^
Weight (kg)	68.70 ± 14.09	73.98 ± 14.76	63.79 ± 11.57	< 0.0001^t^
Height (m)	1.65 ± 0.08	1.71 ± 0.06	1.59 ± 0.06	< 0.0001^MW^
BMI (kg/m^2^)	25.11 ± 4.64	25.20 ± 5.15	25.02 ± 4.18	0.826^MW^
%BF	24.64 ± 8.74	18.87 ± 7.79	30.01 ± 5.63	< 0.0001^t^
%FFM	75.36 ± 8.74	81.12 ± 7.78	69.99 ± 5.63	< 0.0001^MW^

Results are presented as mean ± one standard deviation. *BMI* body mass index (kilograms divided by the square of the height in meters), *BF* body fat, *FFM* free fat mass, *MW* Mann-Whitney, *t t* Student

Airway flow, volume, and pressure variables were significantly greater in men than in women (Table 2). Raw (1.14 ± 0.48 cmH_2_O/L/s) was significantly higher in women (*p* < 0.001) than in men. Airway conductance was significantly higher in men (1.57 ± 0.71 L/s/cmH_2_O) than in women (*p* < 0.001) (Table 2).

**Table 2:** Lung function characteristics of the total sample and by gender

	Total sample	Male	Female	*p* value
Number	83/100	40/48.19	43/51.81	–
FVC (L)	4.46 ± 0.94	5.21 ± 0.69	3.76 ± 0.52	< 0.0001^t^
FEV_1_ (L/s)	3.81 ± 0.78	4.43 ± 0.58	3.24 ± 0.44	< 0.0001^MW^
FEF _25–75%_ (L/s)	4.08 ± 1.00	4.66 ± 1.00	3.54 ± 0.65	< 0.0001^MW^
PEF (L/s)	8.01 ± 1.88	9.53 ± 1.36	6.60 ± 0.97	< 0.0001^MW^
SVC (L)	4.06 ± 0.89	4.70 ± 0.72	3.47 ± 0.56	< 0.0001^t^
ERV (L)	1.33 ± 0.43	1.55 ± 0.45	1.12 ± 0.30	< 0.0001^t^
IC (L)	2.72 ± 0.75	3.14 ± 0.76	2.33 ± 0.47	< 0.0001^t^
RV (L)	1.92 ± 0.71	2.26 ± 0.79	1.60 ± 0.44	< 0.0001^t^
TLC (L)	5.89 ± 1.40	6.79 ± 1.39	5.05 ± 0.72	< 0.0001^MW^
MIP (-cmH_2_O)	100.92 ± 33.91	118.82 ± 33.94	84.30 ± 24.27	< 0.0001^t^
MEP (cmH_2_O)	100.84 ± 27.99	114.85 ± 25.95	87.91 ± 23.42	< 0.0001^t^
Raw (cmH_2_O/L/s)	0.98 ± 0.49	0.81 ± 0.44	1.14 ± 0.48	0.0006^MW^
sRaw (cmH_2_O*s)	3.28 ± 1.25	3.17 ± 1.29	3.38 ± 1.20	0.238^MW^
GAW (L/s/cmH_2_O)	2.45 ± 10.41	1.57 ± 0.71	1.05 ± 0.51	0.0005^MW^
sGAW (1/cmH_2_O*s)	0.09 ± 0.01	0.37 ± 0.13	0.34 ± 0.15	0.164^MW^

*FVC* forced vital capacity, *FEV*_*1*_ volume that has been exhaled at the end of the first second of forced expiration, *FEF*_*25–75*_ forced expiratory flow 25–75%, *PEF* peak expiratory flow, *MIP* maximum inspiratory pressure, *cmH*_*2*_*O* centimeters of water, *MEP* maximum expiratory pressure, *IC* inspiratory capacity, *ERV* expiratory reserve volume, *RV* residual volume, *TLC* total lung capacity, *RAW* airway resistance, *GAW* airway conductance, *sRAW* specific airway resistance, *sGAW* specific airway conductance, *cmH*_*2*_*O/L/s* centimeters of water divided liters divided seconds, *L/s/cmH*_*2*_*O* liters divided seconds divided centimeters of water, *cmH*_*2*_O*s centimeters of water per second, *1/cmH*_*2*_O*s one divided centimeters of water per second, *MW* Mann-Whitney, *t t* Student

Upon analyzing the correlations of lung function variables with BC in the general sample, FEV_1_ and FEF_25–75%_ showed no significant association with BMI or %FFM. A significant inverse relationship was observed (*r* = − 0.540; *p* < 0.0001) between FEV_1_ and %BF. Raw and sRaw showed significant direct associations with BMI and %BF. The highest values were observed for the associations of Raw with BMI (*r* = 0.628; *p* < 0.0001) and %BF (*r* = 0.723; *p* < 0.0001). In men, all BC values showed significant associations with Raw, where the strongest correlation was with %BF (*r* = 0.728). For the sRaw variable, the strongest correlations were with BMI (*r* = 0.617) and %BF (*r* = 0.607). In women, all BC variables showed significant correlations with Raw and sRaw, except for %FFM. Among women, the strongest correlations for both Raw (*r* = 0.615; *p* < 0.0001) and sRaw (*r* = 0.556; *p* < 0.0001) were found with BMI (Table 3).

**Table 3:** Relationship of anthropometric and body composition variables with indicators of airway obstruction in the total sample

	FEV_1_	FEF_25–75%_	Raw(cmH_2_O/L/s)	sRaw(cmH_2_O*s)
Total sample				
BMI (kg/m^2^)	*r* = − 0.044^a^	− 0.038^a^	0.628^a^	0.552^a^
	*p* = 0.688	0.726	< 0.0001	< 0.0001
%BF	*r* = − 0.540^a^	− 0.375^a^	0.723^a^	0.519^a^
	*p* = < 0.0001	0.0005	< 0.0001	< 0.0001
%FFM	*r* = 0.096^a^	0.047^a^	− 0.156^a^	− 0.1507^a^
	*p* = 0.386	0.670	0.157	0.173
Males				
BMI (kg/m^2^)	*r* = − 0.092^a^	− 0.073^a^	0.700^a^	0.617^a^
	*p* = 0.569	0.653	< 0.0001	< 0.0001
%BF	*r* = − 0.087^a^	0.044^a^	0.728^a^	0.607^a^
	*p* = 0.593	0.783	< 0.0001	< 0.0001
%FFM	*r* = − 0.056^a^	− 0.112^a^	− 0.341^a^	− 0.392^a^
	*p* = 0.729	0.490	0.031	0.012
Females				
BMI (kg/m^2^)	*r* = 0.081^b^	0.066^a^	0.574^b^	0.551^b^
	*p* = 0.601	0.674	0.0001	0.0001
%BF	*r* = 0.100^b^	− 0.100^a^	0.4662^b^	0.517^b^
	*p* = 0.520	0.522	0.001	0.0004
%FFM	*r* = 0.136^b^	0.119^a^	− 0.011^b^	0.026^b^
	*p* = 0.383	0.443	0.943	0.867

*BMI* body mass index (kilograms divided by the square of the height in meters), *BF* body fat, *FFM* free fat mass, *FEV*_*1*_ volume that has been exhaled at the end of the first second of forced expiration, *FEF*_*25–75%*_ forced expiratory flow 25–75%; *RAW* airway resistance, *sRAW* specific airway resistance, *cmH*_*2*_*O/L/s* centimeters of water divided liters divided seconds, *cmH*_*2*_O*s centimeters of water per second,

^a^Spearman correlation

^b^Pearson correlation

Regarding the BC and lung function associations, BC variables were adjusted by gender and age (Table 4). It was observed that participants with a BMI over 30 kg/m^2^ showed greater risks of having increased Raw (OR = 26.8) and sRaw (OR = 9.3), regardless of age and gender. Furthermore, individuals with a %BF above p75 had higher risks of greater Raw (OR = 14.04) and sRaw (OR = 4.14). In contrast, it was observed that as %FFM increased, the risk of higher Raw and sRaw diminished, which was observed among women (Table 4).

**Table 4:** Logistic regressions for the association among pulmonary function measured and body composition adjusted by gender and age

	FEV_1_(L)		FEF 25–75% (L/s)		Raw(cmH_2_O/L/s)		sRaw(cmH_2_O*s)	
	OR [95%CI]^a^	*p*	OR [95%CI]^a^	*p*	OR [95%CI]^a^	*p*	OR [95%CI]^a^	*p*
Obese BMI (≥ 30 kg/m^2^)	0.534 [0.102–2.786]	0.456	0.236[0.027–2.031]	0.189	26.837[2.238–321.790]	0.009	9.344[2.218–39.364]	0.002
Gender (female)	1.176 [0.432–3.199]	0.752	0.983[0.356–2.716]	0.974	2.363[0.190–29.388]	0.504	1.312[0.357–4.822]	0.682
Age	1.104 [0.883–1.382]	0.385	1.084[0.862–1.383]	0.492	1.282[0.794–2.071]	0.309	1.105[0.833–1.466]	0.488
Hosmer-Lemeshow	0.288		0.557		0.926		0.329	
Fat mass (%)	0.860 [0.241–3.077]	0.817	0.596[0.149–2.384]	0.465	14.047[1.298–151.961]	0.030	4.144[1.162–14.794]	0.028
Gender (female)	1.215 [0.449–3.290]	0.701	1.038[0.379–2.843]	0.941	1.703[0.159–18.227]	0.660	1.112[0.326–3.787]	0.865
Age	1.094 [0.876–1.367]	0.430	1.068[0.852–1.337]	0.566	1.301[0.819–2.067)	0.265	1.122[0.859–1.465]	0.398
Hosmer-Lemeshow	0.160		0.224		0.850		0.208	
Free fat mass (%)	1.022 [0.947–1.102]	0.581	1.013[0.939–1.094]	0.735	0.770[0.600–0.989]	0.041	0.822[0.727–0.928]	0.002
Gender (female)	1.569 [0.412–5.968]	0.509	1.239[0.325–4.724]	0.754	0.168[0.011–2.615]	0.203	0.143[0.026–0.787]	0.025
Age	1.106 [0.881–1.139]	0.385	1.065[0.847–1.340]	0.588	1.161[0.701–1.923]	0.561	1.047[0.759–1.444]	0.781
Hosmer-Lemeshow	0.452		0.301		0.969		0.346	

*BMI* body mass index (kilograms divided by the square of the height in meters), *OR* odds ratio, *CI* confidence interval, *FEV*_*1*_ volume that has been exhaled at the end of the first second of forced expiration, *FEF*_*25–75%*_ forced expiratory flow 25–75%, *Raw* airway resistance, *sRaw* specific airway resistance, *cmH*_*2*_*O/L/s* centimeters of water divided liters divided seconds, *cmH*_*2*_O*s centimeters of water per second, 75p of Raw male > 1.45, female > 1.86, *sRaw* male-female > 4.76. 25p FEV_1_, male < 4.04, female < 3.09. FEF_25–75%_, male < 3.98, female 3.02

## Discussion

The present study reported the association of BC with AFL in small and medium-gauge airways by measuring FEV_1_, FEF_25–75%_, Raw, and sRaw. The principal result was the inverse association of FEV_1_ and FEF_25–75%_ with %BF. The direct relationships of Raw and sRaw with BMI, and Raw and sRaw with %BF were independent of gender. Furthermore, in men, an inverse association between Raw, sRaw, and %FFM was found. It was also observed that obese patients with higher %BF had a higher risk of presenting with Raw and sRaw regardless of age or gender. Finally, women with a higher %FFM had lower risks for Raw and sRaw, independent of the age variable. Therefore, improving the %FFM would be a protective element of a decrease in Raw and sRaw (see Table 4).

The inverse associations of FEV_1_ and FEF_25–75%_ with BMI and %BF are in partial agreement with the results reported by Duarte et al. [25], in which group excess body fat was associated with diminished lung function, evidenced by higher body fat and lower FEV_1_ and FVC, but this phenomenon was not observed in FEV_1_ among males. In the present study, this significant association was lost after dividing the sample by gender, which may be due to both FEV_1_ and FEF_25–75%_ being variables that reflect medium-gauge airway condition; thus, identifying diminished function would be more complicated among people without chronic respiratory backgrounds than among those with respiratory histories.

Direct relationships of Raw and sRaw with BMI and %BF were observed in the present study in the general sample and in both genders. This finding is in agreement with the findings of Van de Kant et al. [26], who evaluated 34 subjects between 23 and 69 years old for the effects of BMI and %BF on distal airway function and inflammation. Their principal results indicated greater Raw in overweight/obese subjects, which was negatively associated with %BF [26]. The present study added age and gender variables, and worst lung function was observed (p25), which allowed for analyzing ventilatory variables adjusting for age and gender. Regarding Raw and sRaw, body fat distribution might help explain this phenomenon due to the repercussions on breathing mechanics generated by its accumulation in the thorax and/or abdomen [1, 13].

Available information indicates that obesity has been shown to have a direct relation with lung function changes. Fat deposits in the mediastinum, abdomen, and thoracic cavities alter breathing patterns, diminishing thoracic wall compliance [1, 6, 27], which increases intraabdominal and pleural pressure, restricting diaphragm and thoracic wall movement. All of these factors result in reduced expiratory reserve volume (ERV) and residual functional capacity (RFC) [6, 27]. Diminished parenchymal tension over the airway also occurs and mainly affects smaller gauges [27]. Furthermore, long-term obesity can affect lung growth, which has been observed in obese children with signs of dysanapsis or dissociation of growth between airways and lung size. Another disorder associated with people with excess body fat is increased levels of proinflammatory adipokines and cytokines in systemic circulation, which is associated with increased airway inflammation [6, 28].

Finally, the association between %FFM and lung function continues to be studied. In this context, Park et al. [29] studied the effects of the fat-free mass index (FFMI) in men and women 45 ± 13 years of age. They found a direct relationship between FEV_1_ and FFMI in men and women [29]. The results of the present study do not show this association; however, Raw and sRaw showed a negative correlation with %FFM in men. This could be due to (i) the sample studied was younger meaning they could be in an initial phase of the disorder, that is to say, a decrease in cross-sectional area of the smaller caliber airways, (ii) the established inclusion and exclusion criteria allowed us to evaluate apparently healthy nonsmoking participants, (iii) this study assessed the association of %FFM with lung function, whereas the association of an index and lung function was proposed by Park et al. [29], and (iv) MIP and MEP, in individuals of both genders, were found to be within normal ranges, reinforcing the fact that increased Raw and sRaw would be solely due to fat tissue and not diminished compliance associated with reduced muscle force. The gender difference would be explained by the different distribution of fat deposits between men and women. In men, fat accumulation is mainly abdominal, while in women, it mainly occurs in the buttocks and thighs [9]. This is important if we consider the Wilson model of three compartments, where the abdominal cavity is associated with the lower thorax and can therefore positively or negatively affect diaphragmatic activity through the abdominal wall [30]. The role of the latter on the ventilatory pump has been described in physical models as a passive agent that would impact concentric diaphragmatic contraction [31]. However, when the conditions of this compartment are abnormal, which is the case in obesity, ventilatory mechanics change, increasing the possibility of high Raw and sRaw. Thus, one of the recommendations arising from the results found here would be adopting healthy lifestyle habits to control fat tissue.

This study has limitations that must be stated. The reported results are applicable only to the evaluated group and cannot be generalized. Despite this, the concordances with other authors make the data about Raw and sRaw relevant. Secondly, due to the nature of the study, it is not possible to determine causality; future longitudinal studies will be necessary to determine causality. A third limitation is that the groups studied are young adults without smoking habits. However, despite the inclusion and exclusion criteria, significant associations between BC and AFL were observed. In summary, increased %BF is associated with increased AFL in small-gauge airways. Increased %FFM is also associated with a lower risk of increased Raw and sRaw in females.
